# A Whole Genome Assembly of the Horn Fly, *Haematobia irritans*, and Prediction of Genes with Roles in Metabolism and Sex Determination

**DOI:** 10.1534/g3.118.200154

**Published:** 2018-04-05

**Authors:** Kranti Konganti, Felix D. Guerrero, Faye Schilkey, Peter Ngam, Jennifer L. Jacobi, Pooja E. Umale, Adalberto A. Perez de Leon, David W. Threadgill

**Affiliations:** * Texas A&M Institute for Genome Sciences and Society, Texas A&M University, College Station, Texas 77845; † USDA-ARS Knipling-Bushland US Livestock Insects Research Laboratory and Veterinary Pest Genomics Center, Kerrville, Texas 78028; ‡ National Center for Genome Resources, Santa Fe, New Mexico 87505; § Department of Immunology, University of Texas NGS Clinical Laboratory, University of Texas Southwestern Medical Center, Dallas, Texas 75390; ** Arbor Diagnostics Inc., Dallas, Texas 75229; †† Department of Veterinary Pathobiology, Texas A&M University, College Station, Texas 77845; ‡‡ Department of Molecular and Cellular Medicine, Texas A&M University, College Station, Texas 77845

**Keywords:** horn fly, sex determination genes, pesticide resistance, metabolic resistance

## Abstract

*Haematobia irritans*, commonly known as the horn fly, is a globally distributed blood-feeding pest of cattle that is responsible for significant economic losses to cattle producers. Chemical insecticides are the primary means for controlling this pest but problems with insecticide resistance have become common in the horn fly. To provide a foundation for identification of genomic loci for insecticide resistance and for discovery of new control technology, we report the sequencing, assembly, and annotation of the horn fly genome. The assembled genome is 1.14 Gb, comprising 76,616 scaffolds with N50 scaffold length of 23 Kb. Using RNA-Seq data, we have predicted 34,413 gene models of which 19,185 have been assigned functional annotations. Comparative genomics analysis with the Dipteran flies *Musca domestica* L., *Drosophila melanogaster*, and *Lucilia cuprina*, show that the horn fly is most closely related to *M. domestica*, sharing 8,748 orthologous clusters followed by *D. melanogaster* and *L. cuprina*, sharing 7,582 and 7,490 orthologous clusters respectively. We also identified a gene locus for the sodium channel protein in which mutations have been previously reported that confers target site resistance to the most common class of pesticides used in fly control. Additionally, we identified 276 genomic loci encoding members of metabolic enzyme gene families such as cytochrome P450s, esterases and glutathione S-transferases, and several genes orthologous to sex determination pathway genes in other Dipteran species.

The horn fly, *Haematobia irritans*, is one of the most widespread and economically important ectoparasites of cattle in the world. The fly is found in Europe, Africa, Asia, and the Americas, imparting significant discomfort to cattle and economic impact to cattle producers. The entire life cycle of the horn fly is centered upon cattle, as eggs are laid in fresh manure wherein larvae and pupae develop, after which the adult fly emerges and immediately begins feeding upon a bovine host. The adult fly is hematophageous, feeding 20-40 times per day, primarily upon cattle. Economic impact of the horn fly has been estimated at US$2.5 billion annual losses in Brazil alone (Grisi *et al.* 2014). Control of this pest relies upon the use of insecticides, but resistance to the most commonly available products in the pyrethroid and organophosphate classes is a major problem leading to control failures (Guerrero and Barros 2006; Jamroz *et al.* 1998; Barros *et al.* 2001). The effects of climate change and global warming have been predicted to increase the fly’s economic impact upon the U. S. cattle and the cattle industry, particularly in the Northern states (Schmidtmann and Miller 1989). The problem of resistance is driving the search for new control technologies, including vaccine-based approaches (Cupp *et al.* 2004) and female-specific conditional lethality systems (Guerrero *et al.* 2009).

Transcriptomic and genomic information would facilitate research into genes that may contribute to insecticide resistance and development of new control methods, but a sequenced and assembled horn fly genome is not currently available. The only genome sequencing project registered with the National Center for Biotechnology Information (NCBI; Accession PRJNA30967) contains the incipient 454-based transcriptomic data previously reported by our research group (Guerrero *et al.* 2008; Guerrero *et al.* 2009). Additional Sanger-based expressed sequence tag (EST) transcriptome data has been reported by Guerrero *et al.* (2004) and Torres *et al.* (2011). However, these datasets are not comprehensive, as the largest adult fly transcriptome reports only 992 unigenes (Torres *et al.* 2011) and the number of reported unigenes from the embryonic and larval life stages are 2,668 and 2,689, respectively (Guerrero *et al.* 2008).

The size of the horn fly genome was estimated by flow cytometry as approximately 1.2 Gb (Picard *et al.* 2012), as compared to the genome sizes of the related Muscidae flies *Musca domestica* and *Stomoxys calcitrans*, which were reported as 1.0 Gb and 1.1 Gb, respectively. Thus, the horn fly appears to have the largest genome of the Muscidae family members reported to date. Little is known about the complexity of the horn fly genome, although Robertson and Lampe (1995) estimated approximately 17,000 copies of the *mariner* family of transposable elements existed in the genome. To address the lack of comprehensive gene information from the horn fly, we sequenced and assembled the genome of the horn fly using a hybrid approach based upon Pacific Biosciences (PacBio) long reads and Illumina derived short reads. To assist with gene prediction and genome annotation, we sequenced the transcriptome of several tissues and life stages of the horn fly. From the gene model predictions and the transcriptome data, we identified genes encoding proteins with putative roles in sex determination and metabolism of xenobiotics that are potential candidates for developing new methods to control horn flies and identifying mechanisms of insecticide resistance.

## Materials and Methods

### DNA Isolation and Sequencing

Newly emerged, unfed adult flies of mixed sex were collected from the Kerrville susceptible *in vitro* reference strain maintained at the USDA-ARS Knipling-Bushland U. S. Livestock Insects Research Laboratory (Kerrville, TX). This closed fly colony has been reared since 1961 at the Kerrville laboratory, with adults feeding upon citrated bovine blood and eggs seeded into a larval/pupal rearing medium mixture of bovine feces and peanut hulls (Schmidt *et al.* 1976). Flies were knocked down with CO_2_, suctioned into a collection tube, and frozen at -80° within 15-30 min of collection.

Genomic DNA was isolated from 1.5 g of flies according to the Sambrook *et al.* (1989) protocol for isolation of very high molecular weight DNA. Briefly, the frozen flies were pulverized in a liquid nitrogen-cooled mortar and pestle, added to an aqueous buffer, followed by RNAse treatment, digestion by proteinase K, phenol extraction, and extended dialysis in 50 mM Tris, 10 mM EDTA, pH 8.0. DNA size and integrity was verified by 0.6% agarose gel electrophoresis and the genomic DNA stored at 4°.

The PacBio sequencing was performed at National Center for Genome Resources (Santa Fe, NM, USA). One 10 kb and two 20 kb insert DNA libraries were prepared according to PacBio protocols. This includes DNA damage repair, end repair, SMRTbell adapter ligation, and an exonuclease step to remove failed ligation products. For the 10 kb protocol preparation, four μg of genomic DNA was sheared to ∼8 kb with the Covaris G-tube according to manufacturer’s instructions (Covaris Inc., Woburn, MA, USA). For the 20 kb protocol, shearing for both libraries was started on the Eppendorf MiniSpin Plus microcentrifuge (Eppendorf Inc., Hauppauge, NY, USA) at 4000 rpm while steadily increasing speed until the material passed through the synthetic ruby of the G-tube. Both libraries were size selected to remove fragments below 6 kb. The 10 kb library was sequenced on 12 SMRTCells using 3-hour movie times with C2 chemistry and P4 polymerase. One 20 kb library was sequenced on four SMRTCells and the other was sequenced on eight SMRTCells. Both 20 kb libraries were sequenced using 3-hour movie times with C3 chemistry and P5 polymerase.

The Illumina-based sequencing was performed at the National Center for Genome Resources (Santa Fe, NM, USA) using the standard Illumina DNA library preparation protocol and the TruSeq DNA Sample Preparation V2 kit (Illumina, CA, USA). One short-insert paired end library and one long-insert paired end library, gel-selecting for 6-12 kbp insert sizes, was synthesized from the horn fly genomic DNA described above. For the mate-pair, or also known as the long-insert paired end library, the Nextera MatePair Library Prep Kit was used (Illumina, CA, USA). Two μg of gDNA was fragmented by tagmentation at 55° for 30 min. The tagmentation process simultaneously fragments and tags the DNA with junction adapter sequence. The resulting fragments then went through a strand displacement process whereby the overhangs were repaired, making them ready for circularization. The Blue Pippin System (Sage Science, MA, USA) was used to size select the final product to a desired size ranged of 6 – 12 kbp. The size selected sample was circularized overnight. During this step, the ends of the DNA fragments are ligated at the junction adapters after which remaining linear DNA fragments are digested by exonuclease treatment, leaving only the circularized DNA. The circularized DNA was fragmented mechanically on the Covaris S2 system (Covaris Inc., MA, USA) to obtain fragment sizes in the 300 – 1000 bp range. The fragmented DNA samples were end-repaired by the addition of the end repair mix and incubation at 30° for 30 min. The polished fragments were phosphorylated by T4 polynucleotide kinase, followed by the addition of a single A nucleotide to the 3′ end by incubating the end-repaired fragments with A-Tailing mix at 37° for 30 min. The fragment-adapter ligation occurred at 30° for 10 min, after which the ligated product was size-selected by gel electrophoresis, the library fragment range was visualized under brief ultraviolet light, and the desired size range of 300-400 bp excised and subjected to a final PCR amplification step of 10 cycles. All amplified libraries were quantitatively and qualitatively assessed by Nanodrop ND-1000 (Thermo Scientific, DE, USA) and DNA bioanalyzer 2100 (Agilent, CA, USA). Both libraries were sequenced as 100 nt paired ends on three lanes of the HiSeq2000. Following sequencing, the raw reads were processed by the Illumina pipeline and further by the NCGR contaminant filtering pipeline to remove adapter dimers, PCR primers, unused indexes, Illumina PhiX control sequences, and other impurities.

### Genome Assembly

Prior to assembling the genome, 460 million paired-end 100 bp HiSeq reads were filtered and trimmed for any sequencing adapters using Trimmomatic (Bolger *et al.* 2014) resulting in 410 million filtered reads. SOAPdenovo2 (Luo *et al.* 2012) was used to perform *de novo* genome assembly of filtered paired-end short reads at *k-mer* sizes 23, 25, 27, 31, 33, 35 and 37, with best assembly achieved at *k-mer* value 35. The Jellyfish algorithm was implemented for *k-mer* counting (Marçais and Kingsford 2011). Uncorrected PacBio reads were used to fill in gaps using PBJelly2 (English *et al.* 2012). Local misassemblies and any detected incorrect bases were corrected and polished using Pilon (Walker *et al.* 2014). Scaffolds less than 500 bp in size were discarded to create a final draft assembly (Cruz *et al.* 2016).

### RNA Isolation, Sequencing and Expression Quantification

To study the expression patterns of the genes predicted from our assembly, we collected tissues from 16 various life stages and dissected organs of the Kerrville strain of the horn fly. We used eggs collected within 30 min of oviposition, and eggs collected 2-hour, 4-hour, and 9-hour post-oviposition; 1 and 3 day(s) old pupae; unfed adult females and adult females blood feeding for 2, 4, and 24 hr; testes dissected from blood feeding adult males; and ovaries, Malpighian tubules, guts, legs, and salivary glands dissected from blood feeding adult females. RNA was isolated from each of these tissues using the ToTALLY RNA Isolation Kit following the manufacturer’s protocols, including the optional LiCl step to selectively precipitate RNA (Thermo Fisher Scientific, Waltham, MA, USA). DNAse treatment using the Turbo DNA-*free* kit and protocols (Thermo Fisher Scientific) resulted in DNA-free RNA. Integrity and purity of the RNA was verified by agarose gel electrophoresis.

Sequencing was performed at National Center for Genome Resources (NCGR, Santa Fe, NM, USA) using standard RNA-Seq library preparation protocols using 2 lanes of the HiSequation 2000 and the 2 × 100 approach, multiplexing RNAs from 9 and 7 tissues in the 2 lanes. Quality control (QC) was performed on all the samples at NCGR upon receipt and all samples passed. RNA-Seq libraries were prepared and bar-coded using the Maestro-based TruSeq RNA application on the Sciclone NGS platform (PerkinElmer, MA, USA), a robotics system developed and validated for automated library preparation. The Maestro-based TruSeq RNA Application provided a pre-programmed solution for Illumina TruSeq RNA Sample Preparation v2 protocol Part# 15026495 using TruSeq RNA Library Preparation Kit v2, Set A, Part# RS-122-2001 (Illumina, San Diego, CA). Approximately 2 μg of total RNA was used as starting material for most samples, while for samples with limited amounts, only 0.1-1 μg input was used. Total RNA concentration was measured using Qubit RNA HS Assay Kit (Invitrogen, Q32852) on a Qubit 1.0 fluorometer (Invitrogen, CA, USA) and total RNA integrity was checked using the Agilent RNA 6000 Nano Kit (Agilent Technologies, 5067-1511) on a 2100 Bioanalyzer (Agilent Technologies, CA, USA). In the first step of the library preparation workflow, poly-A RNA was purified using oligo-dT attached magnetic beads. Following purification, the mRNA was fragmented into small pieces using divalent cations under elevated temperature. The cleaved RNA fragments were copied into first strand cDNA using reverse transcriptase and random primers. Second strand cDNA synthesis followed, using DNA Polymerase I and RNase H. The cDNA fragments then went through an end repair process, the addition of a single ‘A’ nucleotide, and ligation of the appropriate indexed adapters. These products were purified and amplified by PCR to create the final paired-end cDNA libraries. Library QC was performed by measuring the average size of library fragments using the 2100 Bioanalyzer (Agilent Technologies, CA, USA). Total concentration of DNA was estimated by UV-Vis spectrophotometer Nanodrop 1000 (Thermo Scientific, MA, USA). Yield and efficiency of the adaptor ligation process was measured with a quantitative PCR assay on a 7900 Fast Real-Time PCR System (Applied Biosystems, CA, USA) using primers specific to the adaptor sequence. Libraries that showed adapter dimers at < 200bp peak size were removed via size-selection using the Blue Pippin (Sage Science, MA, USA).

Library fragments at an average size of 300-400 bp were loaded onto an Illumina paired-end flow cell for clustering on the cBot using the instrument specific clustering protocol. Finally, sequencing was performed on the HiSequation 2000 instrument (Illumina, San Diego, CA) with 2x100 nt read-length configuration (100 nt paired-end) using TruSeq SBS Kit v3 – HS. Following sequencing, the raw reads were de-multiplexed and processed by the Illumina pipeline and further by the NCGR contaminant filtering pipeline to remove adapter dimers, PCR primers, unused indexes, Illumina PhiX control sequences, and other impurities. Following these steps, 350 million 100 bp paired-end reads remained and were checked for adapter sequences and low quality bases using Trimmomatic (Bolger *et al.* 2014). Final output resulted in 319 million filtered reads. RNA-Seq reads were aligned to the *de novo* genome assembly using HISAT2 version 2.0.5 (Kim *et al.* 2015). HTSeq (Anders *et al.* 2015) was used to generate raw read counts per gene using intersection-nonempty parameter to account for ambiguous read mappings. Differential gene expression tests were performed using DESeq2 (Love *et al.* 2014) following recommended guidelines by the authors. Plots were generated using R programming language.

### Gene Prediction and Annotation

Prior to the gene prediction step, soft masking of the *de novo* genome assembly was done with RepeatMasker (Smit, A.F.A., Hubley, R. & Green, P. *RepeatMasker Open-4.0* 2008–2015, http://www.repeatmasker.org/) using *D. melanogaster* as reference species, resulting in approximately 3.2% (Supplementary table S1) of the genome being masked. BRAKER1 (Hoff *et al.* 2016) was used for gene prediction which uses GeneMark-ET (Lomsadze *et al.* 2014) to generate initial *ab initio* gene predictions and further trains AUGUSTUS (Stanke *et al.* 2008) to produce a final set of gene models. Although BRAKER1 has been shown to perform equally well with masked and unmasked genome sequence, we used the soft masked genome to prevent any corrupt gene predictions (Hoff *et al.* 2016). Trinotate version 3.0.2 (https://trinotate.github.io/) and InterProScan version 5.21-60.0 (Jones *et al.* 2014) were used to functionally annotate the predicted gene models. Transcript sequences in FASTA format were created using the gene model information from BRAKER1 using gff2fasta (http//github.com/minillinim/gff2fasta) tool. Gene model structure was evaluated using Eval (Keibler and Brent 2003) and genome completeness was evaluated with BUSCO (Simão *et al.* 2015). Gene Ontology (terms) assigned to predicted transcripts and GO plots were produced using WEGO (http://wego.genomics.org.cn).

### Comparative Genomics

Comparative genomic analysis was performed using OrthoVenn (Wang *et al.* 2015) with default cut-off values, which uses a modified version of the OrthoMCL (Li *et al.* 2003) algorithm for identification of orthologous gene clusters based on sequence homology. *M. domestica* proteins were downloaded from the NCBI reference sequence (RefSeq) database (Pruitt *et al.* 2007; ftp://ftp.ncbi.nlm.nih.gov/genomes/Musca_domestica/protein/protein.fa.gz). Peptide sequences for *D. melanogaster* and *Lucilia cuprina* were downloaded from FlyBase (Revision 6.15, ftp://flybase.net/genomes/Drosophila_melanogaster/dmel_r6.15_FB2017_02) and ENSEMBL (Release 35, ftp://ftp.ensemblgenomes.org/pub/metazoa/release-35/fasta/lucilia_cuprina/pep), respectively. Additional sequence homology searches were performed using algorithms from BLAST+ suite version 2.6.0 (Camacho *et al.* 2009). Multiple sequence alignment was performed using MUSCLE version 3.8.1 (Edgar 2004) and a neighbor-joining tree was created using CLC Genomics Workbench 8.0.2 (https://www.qiagenbioinformatics.com/) with Kimura substitution model and 2000 bootstrap replicates. The phylogenetic tree image was generated using iTOL version 3 (Letunic and Bork 2016). Gene model tracks (Figure 3; Figure 5) were generated using GenomeTools (Gremme *et al.* 2013).

### Data Availability

The raw Illumina and PacBio reads from the genomic DNA sequencing were submitted to the National Center for Biotechnology Information (NCBI) Short Read Archive (SRA) database under the BioProject PRJNA30967. The raw Illumina RNA-Seq reads are available at SRA accession number SRR6231656. The raw PacBio reads are available at SRA accession number SRR6231657. The horn fly genome assembly has been deposited at NCBI under the accession PGFW000000000. The version described in this paper is version PGFW010000000. Supplemental material available at Figshare: https://doi.org/10.25387/g3.6057452.

## Results and Discussion

### Sequencing and Genome Assembly

**Figure 1:**
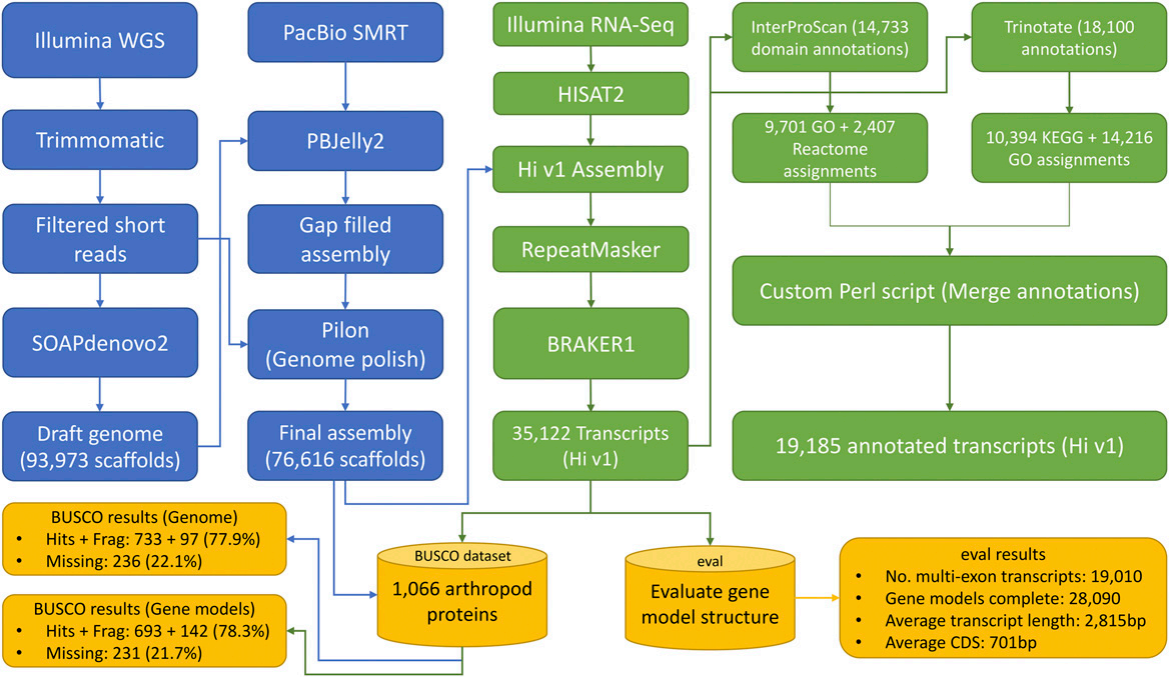
Overall strategy for *de novo* assembly and annotation. Filtered Illumina short reads were assembled using SOAPdenovo2. Subsequently, gaps were filled with uncorrected PacBio reads using PBJelly2 and the resulting genome assembly was polished using Pilon. BRAKER1 was used to predict gene models and InterProScan and Trinotate were used to generate combined gene annotation for the predicted gene models.

The overall assembly and annotation pipeline is shown in Figure 1. A total of 85 billion (71X coverage) Illumina filtered bases and 12 billion (10X coverage) uncorrected bases from 24 PacBio SMRT cells were used to perform *de novo* assembly. PacBio SMRT long reads are estimated to have an error rate of approximately 15% and it has become a general practice to consider error correcting PacBio reads using reads generated from a less error-prone second generation sequence technology such as Illumina. However, it has also been shown that using this type of error correction to achieve a final error rate of approximately 1% results in loss of approximately 18–70% of sequenced bases (Chin *et al.* 2013; Salmela and Rivals 2014; Hayan *et al.* 2014) and most of the self-correction tools for PacBio reads require at least >50X coverage of long reads (Lin and Liao 2015). On the other hand, some *de novo* assembly projects have shown a 10-fold improvement in N50 contig size and a much less fractured scaffold structure by using uncorrected PacBio reads for gap filling and joining contigs (Powers *et al.* 2013). Considering the coverage requirements and sequencing data that was available, with our goal being to generate the best possible contiguous and complete genome assembly, we tried both approaches. We initially used proovread (Hackl *et al.* 2014) to error correct PacBio reads prior to assembly. This resulted in a loss of approximately 62% of sequenced PacBio bases (Supplementary table S2). For comparison, we used all the available PacBio read information without error correction for gap filling and connecting contigs. We found that the final assembly with this approach had a lower number of gaps when we used uncorrected PacBio reads (Table 1, scaffold %N = 47.22%) compared to using the proovread error corrected PacBio reads (68%, Supplementary table S2). We used Pilon for genome polishing and for correcting any local misassemblies and ambiguous bases. Pilon analysis confirmed that 62% of the sequenced PacBio bases are not covered by Illumina short reads, thus the proovread approach would discard those uncorrected PacBio bases and not use them for assembly and scaffolding (Supplementary table S2). Therefore, the assembly we report herein uses all PacBio reads for gap filling and connecting contigs and scaffolds.

**Table 1:** Genome assembly statistics

**Statistics**	**Hi v1.0**
Number of contigs used in assembly	212,677
Number of resulting scaffolds	76,616
Total nt in scaffolds	1,143,537,531
Longest scaffold (nt)	268,115
Shortest scaffold (nt)	500
Number of scaffolds > 1K nt	69,951
Number of scaffolds > 10K nt	41,647
Number of scaffolds > 100K nt	262
Number of scaffolds > 1M nt	0
Mean scaffold size (nt)	14,926
Median scaffold size (nt)	10,609
N50 scaffold length (nt)	23,099
L50 scaffold count	14,391
scaffold % A	17.78
scaffold % C	8.61
scaffold % G	8.60
scaffold % T	17.78
scaffold % N	47.22

The final polished genome assembly size of 1.143 Gb is approximately 95.4% of the 1.197 Gb *H. irritans* genome size previously estimated (Picard *et al.* 2012) and 91.4% of the size estimated using *k-mer* frequency and depth of coverage (Marçais and Kingsford 2011; Supplementary table S2). The assembly consists of 76,616 scaffolds with N50 scaffold size of 23 kb and the longest scaffold size is 268.1 kb (Table 1). Any scaffolds <500 bp were removed from further downstream analyses. Approximately 47% of the genome is still unfilled, and this may reflect the significant amount of repetitive DNA in this genome (Robertson and Lampe 1995). Encouragingly, genome quality assessment with BUSCO indicated 78% genome completeness based on the 1,066 arthropod ancestral protein set (Supplementary table S3). Among the identified BUSCO hits, 68.8% of them were marked as complete while 9.1% of them were marked as fragmented. Alignment of RNA-Seq reads from the various life stage-specific tissues to the *de novo* genome assembly using HISAT2 yielded an overall mapping rate between 50–60% (Supplementary table S3). Additionally, we downloaded 4,920 *H. irritans* mRNA transcripts / partial CDS from GenBank in FASTA format (query term: https://www.ncbi.nlm.nih.gov/nuccore?term=%22Haematobia%20irritans%22[porgn], Supplementary file S1). Approximately 25% of these transcripts are from adult flies (Torres *et al.* 2011), while most of the remainder are embryo or larval stage transcripts (Guerrero *et al.* 2008). Alignment of these 4,920 downloaded transcript sequences to the *de novo* genome assembly using BLASTn found that 3,060 (62.1%) of these sequences aligned with more than 90% sequence identity over 70% query sequence length (Supplementary table S4), further suggesting that our assembly captures a majority of the horn fly’s protein coding genes.

### Functional Annotation and Comparative Genomics

Using RNA-Seq reads as a sole source of evidence for gene prediction with BRAKER1 has been shown to predict genes more accurately than solely *ab initio* prediction methods (Hoff *et al.* 2016). Using RNA-Seq alignments to our horn fly *de novo* genome assembly as evidence, 34,413 gene models were predicted comprising 35,122 mRNA transcripts that includes 709 alternative transcript isoforms (Table 2). More than 79% (28,091) of the predicted transcripts were considered complete, possessing both a start and a stop codon. The average transcript length, coding region length (CDS), and exon size was 2,815 bp, 701 bp, and 352 bp, respectively (Table 2). BUSCO analysis of this set of BRAKER1 predicted peptide sequences shows similar results to the BUSCO analysis of our final genome assembly (78% completeness), with 65.0% marked as complete and 13.3% marked as fragmented (Supplementary table S3). The slight increase in the fragmented BUSCO hits may be due to a reduced ability of BRAKER1 to construct full length gene models in genome regions containing relatively low coverage of RNA-Seq reads.

**Table 2:** Gene model structure

**Statistics**	**Hi v1.0**
Transcripts per gene	1.02
Total number of gene models	34,413
Total number of transcripts	35,122
Alternative isoforms	709
Average transcript length (nt)	2,815
Average transcript coding length (nt)	701
Number of complete transcripts	28,090
Number of single-exon transcripts	15,402
Single-exon transcript average length (nt)	486
Number of total exons	66,976
Average exon length	352

Databases that compile signatures diagnostic for protein families, domains or functional sites are important tools for the computational functional classification of newly determined sequences that may lack *in vivo* or biochemical characterization (Apweiler *et al.* 2001). To help annotate the *H. irritans* genome, we used InterProScan with all the available signature database searches enabled. Table 3 shows the summarized results, wherein a total of 14,733 unique predicted transcripts have at least one annotation from one of the signature databases. Since each of the protein signature databases uses its own threshold cutoff values, annotations were only assigned if there was an InterProScan accession id associated with the hit. In parallel, we were able to assign annotations to 18,100 unique predicted transcripts using Trinotate (Table 3). Annotation sources from Trinotate include BLASTx and BLASTp to find top hits from the SwissProt protein sequence database (Bairoch and Apweiler 2000), SignalP and TMHMM to identify signal peptide cleavage sites and transmembrane domains, and eggNOG (evolutionary genealogy of genes Non-supervised Orthologous Groups) to identify gene orthologies. Trinotate annotations indicate the presence of 3,274 transmembrane domains (TMHMM) and 1,598 signal peptide cleavage sites (SignalP) (Table 3, Supplementary table S5). The number of TMHMM-predicted alpha helices ranged from 1 to 18 per transcript. The eggNOG analysis reveals identification of 10,386 orthologous families associated with 3,484 unique NOG IDs. Finally, a custom Perl script (Supplementary file S2) was used to combine all annotations from both InterProScan and Trinotate, resulting in a total of 19,185 annotated transcripts (Figure 1). We used the following gene nomenclature to create gene names: LOCUS_ORGANISM_ASSEMBLYVERSION_GENE.TRANSCRIPTISOFORM (ex: LOC_Hi_v1_g7554.t2). These functional annotation assignments can be found in Supplementary file S3.

**Table 3:** Functional annotation assignments of predicted transcripts in the *H. irritans* genome by various programs

**Statistics**	**Hi v1.0**
**InterProScan**	
CDD	3,900
Coils	3,540
Gene3D	9,967
Hamap	115
MobiDBLite	13,164
Pfam	12,571
PIRSF	300
PRINTS	1,932
ProDom	90
ProSitePatterns	2,506
ProSiteProfiles	6,013
SFLD	18
SMART	4,533
SUPERFAMILY	10,674
TIGRFAM	379
GO	9,701
Reactome	2,407
**Trinotate**	
BLAST	13,622
Pfam	13,031
SignalP	1,598
TMHMM	3,274
eggNOG	10,386
KEGG	10,934
GO	14,650

**Figure 2:**
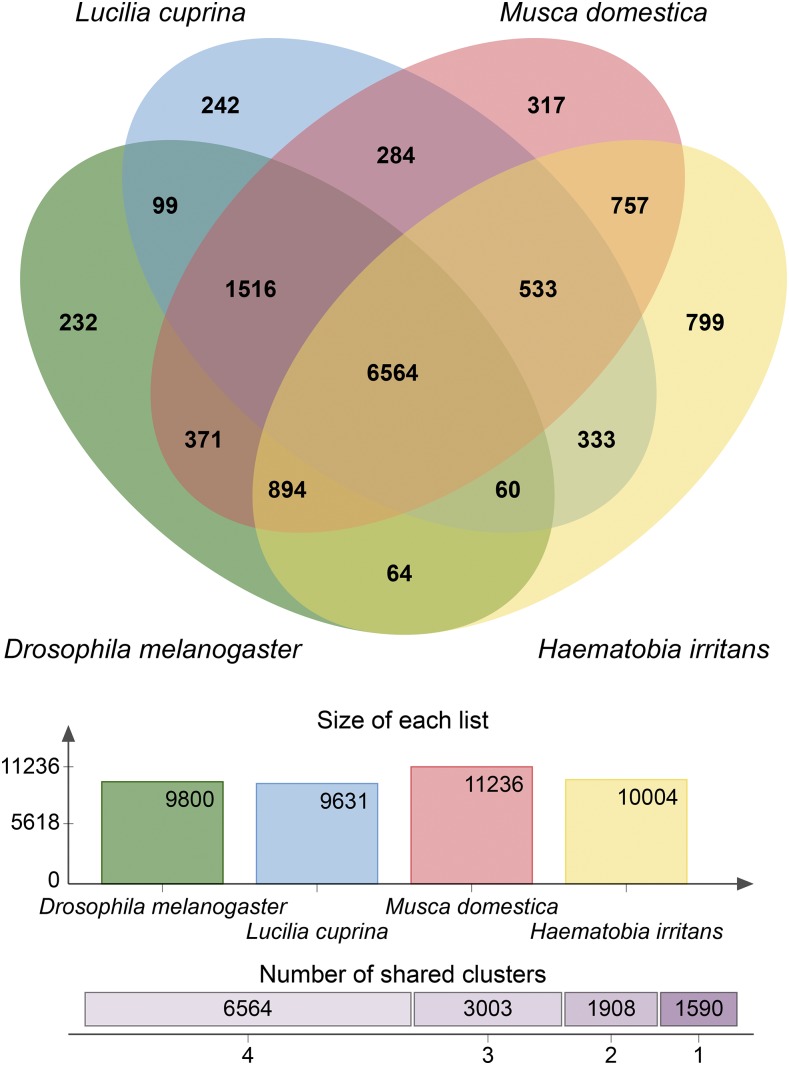
Venn diagram showing orthologous clusters shared between 4 Dipteran species. OrthoVenn was used to generate the diagram comparing the occurrences of orthologous clusters in 3 fly species, *D. melanogaster* (green), *L. cuprina* (blue), and *M. domestica* (red), to *H. irritans* (yellow). The total number of clusters found in each species is shown directly below the Venn diagram. The number of clusters that were shared between 2, 3, and 4 of the species was 1,908, 3,003 and 6,564 respectively. There were 1,590 clusters that were found in only 1 of the 4 species (clusters of singleton genes).

Using the available proteomes of *D. melanogaster*, *L. cuprina*, *M. domestica*, and *H. irritans*, we identified 6,564 orthologous clusters shared between all 4 species (Figure 2). Single copy orthologous clusters contain only a single copy of the protein in each of the species examined. These types of proteins have maintained their single copy status throughout the time after species divergence. We identified 3,699 single copy clusters (Supplementary table S6). These flies are all in the Diptera Order, but are classified in different families. There were 799, 317, 242, and 232 clusters unique to *H. irritans*, *M. domestica*, *L. cuprina*, and *D. melanogaster*, respectively. It is unknown why the horn fly has more unique clusters than the other species analyzed. Perhaps this is due to the horn fly’s characteristic of having a blood-feeding adult life stage and the egg, larval, and pupal stages all being solely dependent on a bovine host. The life cycles of the other 3 species do not revolve around a single host and can be generalists regarding host selection for feeding and development. *D. melanogaster* parasitizes and develops within a large variety of fruits while *M. domestica* and *L. cuprina* develop in dead and decaying matter of various origins. Further inspection of putative annotations of the 799 protein clusters specific to *H. irritans* shows that a large number of inparalogs (1,362) belong to the *mariner* family of transposable elements (Supplementary table S7), implying a high level of activity for *mariner* in the horn fly and congruent with findings by Robertson and Lampe (1995). RepeatMasker analysis is also confirmatory, identifying 36,479 *IS630/Mariner/Tc1/Pogo* class of DNA transposons that accounts for 1.23% of the genome (Supplementary table S1). Transposable elements can have dramatic effects upon gene expression by integration directly into a gene coding region or a gene regulatory region. Further study of these DNA transposons would be interesting to examine how transposition events might be directly impacting the evolution of the horn fly, perhaps facilitating survival in the presence of human imposed stresses such as insecticide usage or advancing warming due to climate change. Additionally, 337 of the 799 horn fly-unique clusters have informative annotations. Of these 337, approximately 50% are related in some fashion to transposons. *H. irritans* shared 8,748, 7,490, and 7,582 clusters with *M. domestica*, *L. cuprina*, and *D. melanogaster*, respectively (Figure 2), reflective of the inter-family relationship that horn fly has with *M. domestica*. Interestingly, despite belonging to the Calliphoridae Family, *L. cuprina* shares 8,899 clusters with the Muscidae Family member *M. domestica*. This is almost as many as the 8,748 that are shared between the two Muscidae, *H. irritans* and *M. domestica*.

### Gene Ontology

The Gene Ontology (GO) assignments from both Trinotate and InterProScan analyses were merged using a custom Perl script (Supplementary file S2) resulting in a total of 14,851 transcripts with GO annotations (Table 3; Supplementary table S8; Supplementary figure S1). The most abundant biological processes in the *H. irritans* v1.0 predicted transcript set were cellular process (72.5%), metabolic process (61.2%) and biological regulation (41.6%) (Supplementary table S8). The most abundant molecular functions were binding (75.4%) and catalytic activity (46.8%). The most abundant cellular components are cell (71.1%) and cell part (71%). It is quite interesting that approximately 9,093 transcripts were annotated with the metabolic processes GO term, underscoring the importance of this category of fly genes that includes those with detoxification function discussed below. These GO term annotations to specific predicted gene models are included in Supplementary file S3.

### Pesticide Resistance and Metabolism/Detoxification Genes

**Figure 3:**
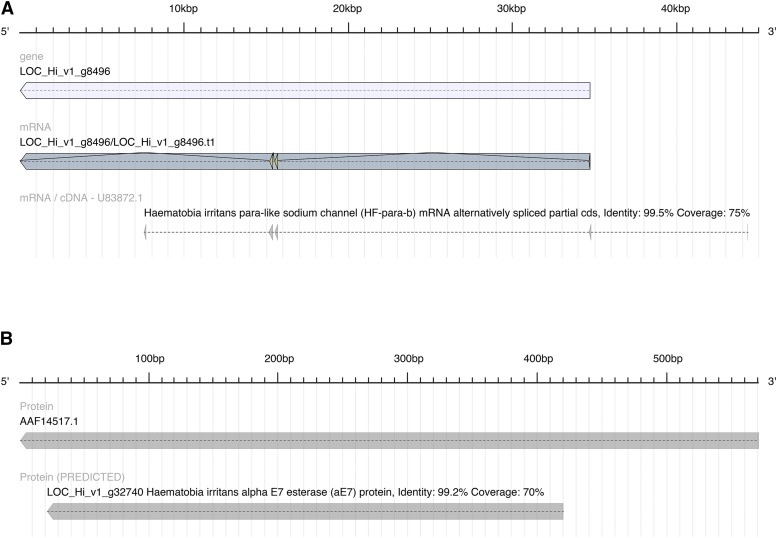
A: Sodium channel gene model prediction. Gene model LOC_Hi_v1_g8496 was predicted to function as a sodium channel protein and the high-scoring segment pairs (HSPs) from the BLASTn analysis are shown. B: Peptide sequence of predicted transcript LOC_Hi_v1_g32740.t1 aligning to previously reported complete CDS sequence of *H. irritans*’ alpha E7 esterase (aE7).

Target site- and metabolism-based insecticide resistance are important mechanisms that lead to horn fly control problems for cattle producers. We searched the assembled genome and transcriptome to identify candidates for insecticide resistance-associated genes. Pyrethroid class insecticides target sodium channels, and two horn fly sodium channel gene mutations have been identified that lead to target site pyrethroid resistance (Guerrero *et al.* 1997). The full-length sodium channel coding region has not been determined in the horn fly, so we used BLASTn and a partial cDNA sequence from the horn fly sodium channel coding region (NCBI Accession No. U83872) and identified a novel gene locus aligning with greater than 99% sequence identity over 75% of the cDNA query sequence length (Figure 3A). This locus is LOC_Hi_v1_g8496 on Contig29567 and information on the annotation (Supplementary file S3), DNA sequence (Supplementary file S4), and translation product (Supplementary file S5) is available. This 44,357 bp partial transcript isoform (LOC_Hi_v1_g8496.t1) contains 3 exons from the sodium channel protein coding region and lacks a stop codon according to the prediction made by BRAKER1. Our previous research had identified at least four introns in this region of the sodium channel gene and the genomic sequence from this locus will assist our investigations into possible alternative splicing and differential expression of this important gene (F. Guerrero, unpublished data).

Metabolism-based pesticide resistance can develop through actions of enzymes capable of detoxifying or sequestering xenobiotics, particularly those enzyme families known as cytochrome P450, esterase, and glutathione S-transferase. Metabolic resistant populations of horn flies have been documented (Sheppard 1995; Guerrero and Barros 2006) and we sought to identify members of these gene families in the *H. irritans* genome assembly v1.0. We identified 283 transcripts with functional annotations related to these metabolic enzyme families, with 165, 99, and 19 belonging to cytochrome P450s, esterases, and glutathione S-transferases, respectively (Supplementary table S9).

Of the 165 presumptive horn fly cytochrome P450-encoding transcripts, 96.3% (159) are associated with at least one InterProScan protein domain identifier. Over 87% (145) of the cytochrome P450 transcripts could be assigned to the CYP6 (69), CYP4 (50), CYP3 (20) and CYP9 (6) families. Comparison of distribution of these cytochrome P450 families in *M. domestica* (Supplementary figure S2) showed similar patterns in the two species. Cytochrome P450s from these families have been shown to have roles in insecticide resistance of *D. melanogaster* (Daborn *et al.* 2002) and *M. domestica* (Højland *et al.* 2014; Smith and Scott 1997) and we would expect some of these P450 families to also have roles in horn fly insecticide resistance mechanisms. Specifically, we identified 7 transcripts with sequence similarity and annotation to CYP12A2 (Supplementary table S9, highlighted in orange) which has been shown to metabolize a variety of insecticides and xenobiotics in *M. domestica* (Højland *et al.* 2014). As the mosquito also is a blood feeding pest, we looked at some of the pesticide resistance-related P450 genes in this organism. The CYP6AA3 and CYP6P7 genes are overexpressed in a pyrethroid resistance strain of *Anopheles minimus* and modeling of the active site cavities of these enzymes substantiated possible roles in pyrethroid metabolism (Lertkiatmongkol *et al.* 2011). Chandor-Proust *et al.* (2013) show that mosquito P450s from the CYP6Z subfamily can degrade pyrethroid metabolites, implicating this subfamily in pyrethroid resistance mechanisms in *Aedes aegypti*. Ibrahim *et al.* (2016) demonstrated that *CYP6P4* is one of the important resistance mechanisms present in a pyrethroid resistant *Anopheles arabiensis* population from Chad, Africa. Thus, members of the mosquito’s CYP6 family appear to have important roles in pesticide resistance. We expect one or more of the 69 members of the CYP6 family we identified in the horn fly genome will be interesting targets for pesticide resistance research. Ongoing transcriptomics studies are looking at gene expression of specific P450s in pyrethroid and organophosphate resistant horn fly populations (F. Guerrero, unpublished data). The comprehensive nature of the horn fly genome assembly will ensure the entirety of the horn fly’s metabolic capacity can be examined at the transcriptome level.

The 99 predicted members of the esterase family of enzymes include acetylcholinesterase, carboxylesterase, cholinesterase, metallophosphoesterase, methylesterase, pectinacetylesterase, phosphodiesterase, phosphoesterase, phosphotriesterase, thioesterase and thiolesterase (Supplementary table S9). The horn fly alpha-E7 carboxylesterase has been shown to have elevated transcript levels in diazinon resistant field-collected horn flies (NCBI Accession No. AAF14517.1; Guerrero 2000). Orthologs of this gene in *M. domestica* (Claudianos *et al.* 1999) and *L. cuprina* (Newcomb *et al.* 1997) can contain amino acid substitutions in the wild type sequence that are responsible for metabolic resistance to organophosphate insecticides in some fly populations. Using BLASTp and our predicted gene locus transcript LOC_Hi_v1_g32740.t1 as query, we found that LOC_Hi_v1_g32740.t1 aligned with >99% sequence identity and 70% query sequence coverage to AAF14517.1 the alpha-E7 carboxylesterase (Figure 3B; Supplementary file S3; Supplementary file S4; Supplementary file S5). Comparing the predicted gene locus transcript to the cDNA encoding the alpha-E7 carboxylesterase (NCBI Accession No. AF139082.1; Guerrero 2000), there are 5 introns predicted to occur in this gene coding region (Supplementary file S3).

Glutathione S-transferases detoxify insecticides by conjugating glutathione to the active ingredient, facilitating conversion to inactive substances that can be secreted from the cell. Strains of *M. domestica* with glutathione S-transferase-mediated insecticide resistance have been reported, although the focus of that study was upon enzyme activity rather than specific genes (Clark *et al.* 1986). Synergist studies using diethylmaleate, which inhibits glutathione S-transferase activity, have indicated there is a component of metabolic resistance to pyrethroid in some horn fly populations in Texas that appears to be due to glutathione S-transferase activity (Li *et al.* 2009). Our cataloging of the 19 *H. irritans* transcripts with annotations of glutathione S-transferase (Supplementary table S9) is an initiation point for gene-based studies of glutathione S-transferase involvement in horn fly insecticide resistance.

**Figure 4:**
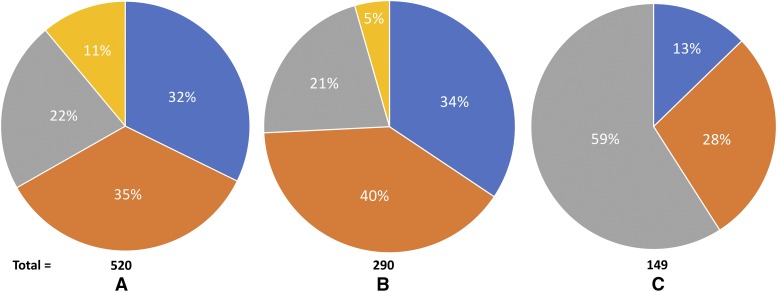
Distribution of metabolic enzyme gene families in 4 Dipteran species. Total numbers of genes for each family are shown below the pie chart for cytochrome P450 (A), esterase (B), and glutathione S-transferase (C). The % value in each colored slice represents the % of the total for that species. Orange: *M. domestica*, blue: *H. irritans*, gray: *D. melanogaster*, yellow: *L. cuprina*.

Comparing the 3 metabolic enzyme families across the 4 Dipteran species, *H. irritans*, *M. domestica*, *D. melanogaster*, and *L. cuprina* (Figure 4) shows that *H. irritans* and *M. domestica* have similarities in numbers of P450s and esterases, with both of those families more abundant than in either *D. melanogaster* or *L. cuprina*. *D. melanogaster* possesses a greater percentage of the glutathione S-transferase family than the other 3 fly species combined (Figure 4C). Looking at the distribution of the metabolic enzyme families within each species (Supplementary table S10; Supplementary figure S3), *H. irritans* has 58.3%, 34.98%, and 6.71% of its metabolic enzymes distributed between the P450, esterase, and glutathione S-transferase families, respectively. For comparison, *M. domestica* has a similar distribution of 53.39%, 34.22%, and 12.39% for P450, esterase, and glutathione S-transferase families, respectively. *D. melanogaster* shows 43.61%, 23.31%, and 33.08% of its metabolic enzymes distributed between the P450, esterase, and glutathione S-transferase families, respectively (Supplementary figure S3). The increased percentage of glutathione S-transferases in *D. melanogaster* is the major difference between the three flies. Interestingly, the initial release of the *M. domestica* genome annotation had 282 genes identified as P450s, esterases, or glutathione S-transferase (Scott *et al.* 2014). Subsequent annotation releases increased that number to the 339 currently shown in Supplementary table S10. It would be expected that the numbers shown for the *L. cuprina* assembly and even our own *H. irritans* assembly v1.0 would change as annotation improves for these two relatively new genome assembly releases.

### Sex Determination Related Genes

**Figure 5:**
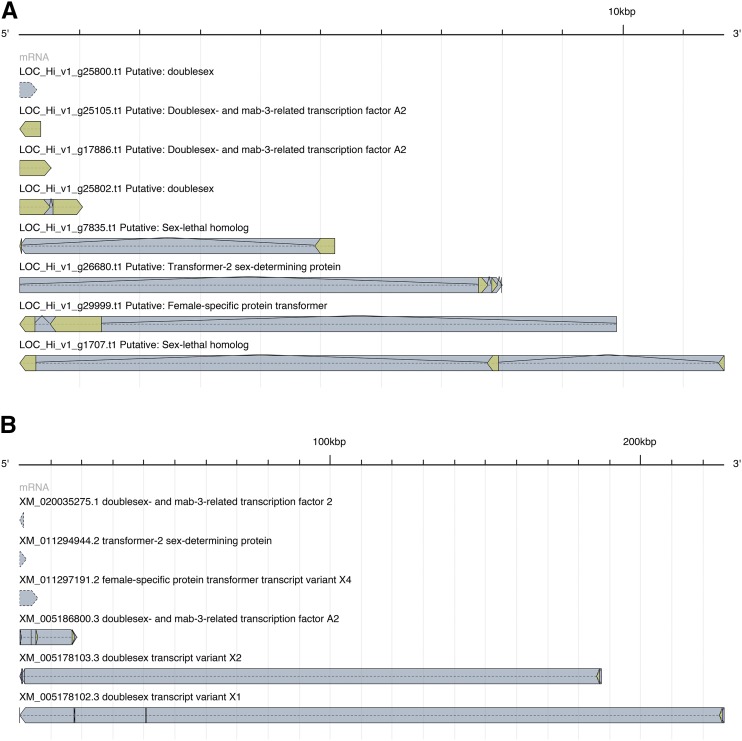
Transcripts with functional annotations related to sex determination. (A): *H. irritans*; (B): *M. domestica*. The scale of the transcript lengths are indicated by hash marks every 1 kbp (A) and 10 kbp (B).

**Figure 6:**
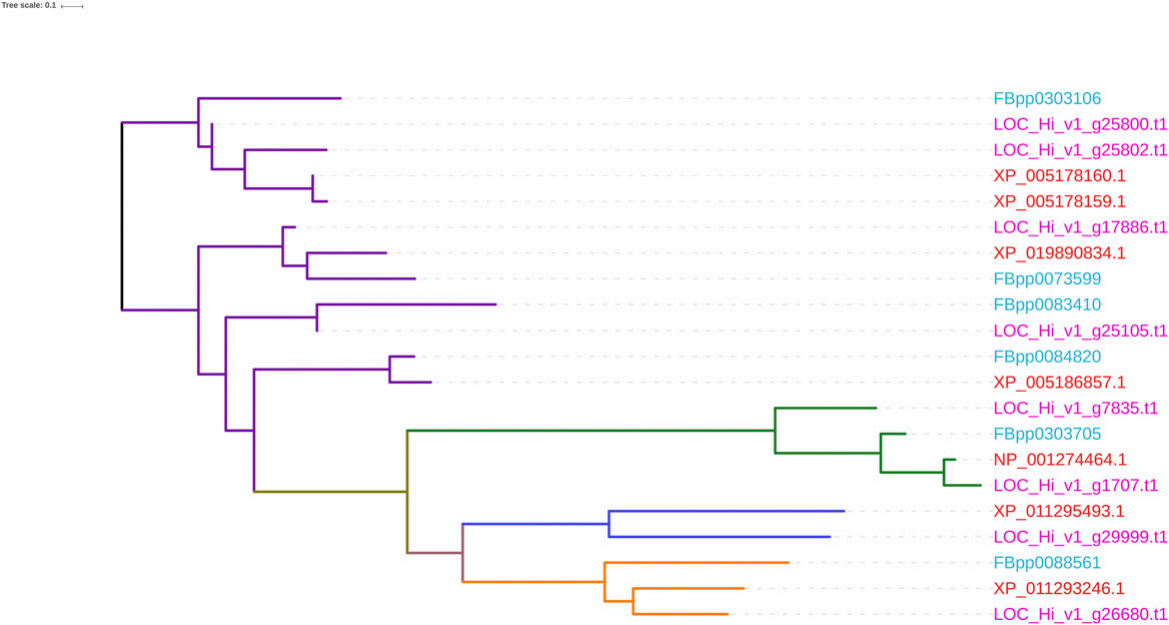
An unrooted tree depicting sex determination-related open reading frames identified in the *H. irritans* genome assembly v1.0. Sex determination proteins, Doublesex (purple colored leaves), Sex-lethal (green leaves), female-specific Transformer (dark blue leaves), and Transformer (orange leaves) from *H. irritans* are compared to their counterparts in *M. domestica* and *D. melanogaster*. Sequence IDs from each species are differentiated by colored text, with *H. irritans* represented in pink, *D. melanogaster* in sky blue, and *M. domestica* in red.

An interesting pest control technology is being pursued in New World screwworm that is based upon the release of male flies containing a dominant female conditional lethal transgene (Concha *et al.* 2016). This transgene makes use of an intron derived from the *transformer* gene of the sex determination pathway of the screwworm. The sex determination pathways used in animals are diverse, although several of the genes along the pathway are conserved (Gempe and Beye 2011). Among these that have been identified in *M. domestica* are *Sex-lethal*, *doublesex*, *transformer*, and *transformer-2* (Dübendorfer *et al.* 2002), and recently a male determining factor has been discovered in this species (Sharma *et al.* 2017). We have identified eight gene loci in our *H. irritans* genome assembly with functional annotations related to sex determination, including *Sex-lethal* (partial protein coding in Contig 28289, LOC_Hi_v1_g7835.t1, partial protein coding in Contig 18898, LOC_Hi_v1_g1707), *doublesex* (partial protein coding in Contig 3052, LOC_Hi_v1_g25800.t1), *transformer* (partial protein coding in Contig 7300, LOC_Hi_v1_g29999.t1), and *transformer-2* (partial protein coding in Contig 4109, LOC_Hi_v1_g26680.t1; Supplementary table S11, Supplementary file S5). A comparison of the gene structures of these sex determination pathway genes from *H. irritans* and *M. domestica* can be seen in Figure 5. Some of the *M. domestica doublesex* variants appear to have much larger introns than the putative *H. irritans* gene. Since the *H. irritans* sex determination pathway has been largely unexplored until now, other variants may remain to be discovered. Phylogenetic tree analysis shows that the *H. irritans* orthologs are closer to their counterparts in *M. domestica* than to those in *D. melanogaster* (Figure 6). However, since sex determination pathways are very diverse, evolutionary relatedness will probably not be a reliable predictor of sex determination pathway in a specific organism.

### Differential Gene Expression

To study patterns of expression of the genes predicted from our assembly, we used RNA-Seq data from 16 tissues and life stages from the horn fly and aligned the reads to the predicted gene models that are listed in File S3. This allowed a view of expression patterns for predicted genes in specific tissues. Relative expression levels of each corresponding horn fly transcript predicted from our assembly are shown in Supplementary table S12. We found 22,698 genes were expressed in at least one tissue. Because our RNA-Seq approach did not consist of replicated samples, quantitative comparisons would not be valid. However, this initial gene expression study can direct future quantitative approaches.

**Figure 7:**
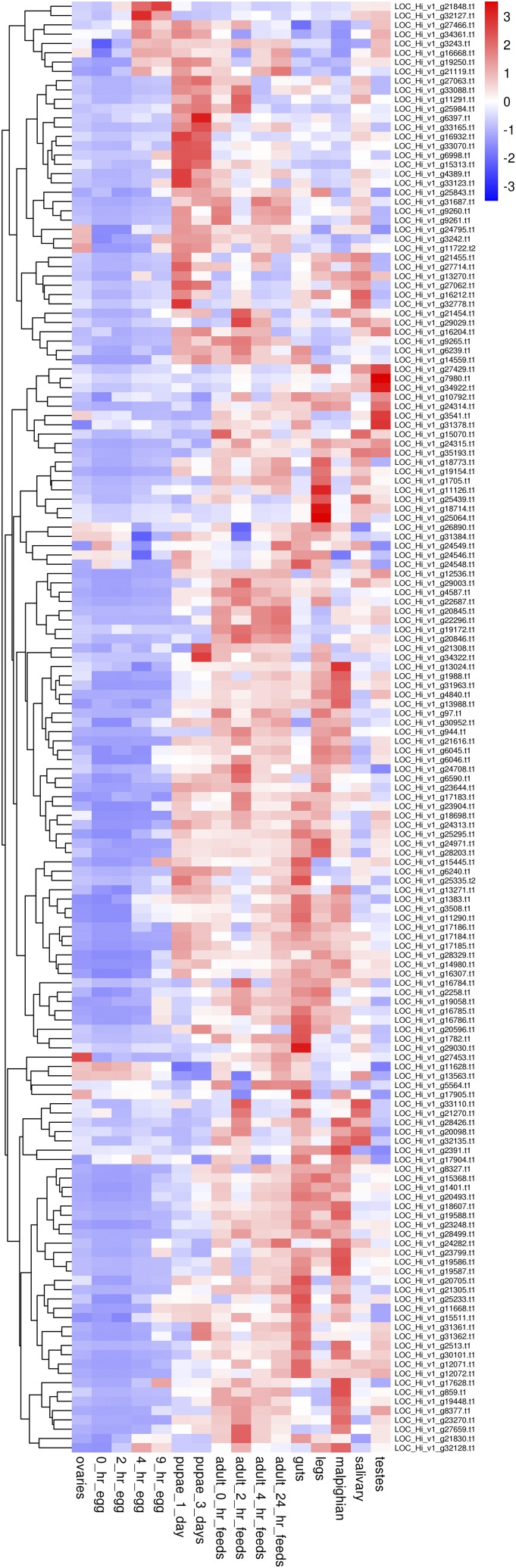
Heat map of relative expression levels of predicted cytochrome P450 gene models across several life stage specific tissues.

Focusing upon 185 predicted transcripts with annotations related to sex determination, cytochrome P450s, and insecticide target sites, we placed the expression pattern data in Supplementary table S13 and produced a heat map expression display from this dataset (Supplementary figure S4). The expression data on the sex determination pathway-related transcripts shows doublesex and transformer express in the pupal and adult stages, while sex-lethal, the transcription factor A2, and transformer-2 express primarily in the eggs. This information will guide future studies aimed at isolation of full-length protein coding regions of the sex determination pathway genes. Expanding upon the data in this Table, we developed a heat map expression display for the cytochrome P450-like transcripts (Figure 7). The figure represents the horn fly’s development from egg to adult, going left to right. It is evident that cytochrome P450-like gene transcription is almost uniformly low in the egg developmental stages compared to the adult stages where the fly is ingesting large volumes of bovine blood and the blood-associated toxic heme. The pupal stage also contains a number of P450-like transcripts whose expression increases at the pupal stage compared to the egg stage. In the adult fly, gut has the greatest overall expression of P450-like transcripts compared to the salivary glands, testes, legs, and malpighian tubules. The predictions of the gene coding regions of these metabolic enzymes coupled with the tissue expression RNA-Seq data will be very useful for determining full length transcripts and their roles in metabolism-based insecticide resistance. The data generated in this survey of tissues and life stages is from an insecticide susceptible laboratory strain and surveys of field populations with varying degrees of resistance should be conducted.

### Conclusions

We have sequenced, assembled and annotated the first genome of the horn fly, *H. irritans*. The size of our v1.0 genome assembly is 1.14 Gb and we have predicted 34,413 gene models using RNA-Seq data as evidence. We have identified genes related to insecticide resistance, detoxification of xenobiotics, and sex determination, which will support new insights into mechanisms of insecticide resistance and new methods for control. Even though the current assembly has ∼47% gaps, BUSCO analysis indicated 78% genome completeness. The availability of this genome should further research programs in horn fly and Dipterans.
